# Encoding of Aversion by Dopamine and the Nucleus Accumbens

**DOI:** 10.3389/fnins.2012.00137

**Published:** 2012-09-25

**Authors:** James E. McCutcheon, Stephanie R. Ebner, Amy L. Loriaux, Mitchell F. Roitman

**Affiliations:** ^1^Department of Psychology, University of Illinois at ChicagoChicago, IL, USA

**Keywords:** voltammetry, electrophysiology, conditioned taste aversion, taste reactivity, reward, ventral tegmental area

## Abstract

Adaptive motivated behavior requires rapid discrimination between beneficial and harmful stimuli. Such discrimination leads to the generation of either an approach or rejection response, as appropriate, and enables organisms to maximize reward and minimize punishment. Classically, the nucleus accumbens (NAc) and the dopamine projection to it are considered an integral part of the brain’s reward circuit, i.e., they direct approach and consumption behaviors and underlie positive reinforcement. This reward-centered framing ignores important evidence about the role of this system in encoding aversive events. One reason for bias toward reward is the difficulty in designing experiments in which animals repeatedly experience punishments; another is the challenge in dissociating the response to an aversive stimulus itself from the reward/relief experienced when an aversive stimulus is terminated. Here, we review studies that employ techniques with sufficient time resolution to measure responses in ventral tegmental area and NAc to aversive stimuli as they are delivered. We also present novel findings showing that the same stimulus – intra-oral infusion of sucrose – has differing effects on NAc shell dopamine release depending on the prior experience. Here, for some rats, sucrose was rendered aversive by explicitly pairing it with malaise in a conditioned taste aversion paradigm. Thereafter, sucrose infusions led to a suppression of dopamine with a similar magnitude and time course to intra-oral infusions of a bitter quinine solution. The results are discussed in the context of regional differences in dopamine signaling and the implications of a pause in phasic dopamine release within the NAc shell. Together with our data, the emerging literature suggests an important role for differential phasic dopamine signaling in aversion vs. reward.

## Introduction

Since Olds and Milner’s (1954) seminal observation that animals will self-administer current to regions of their own brain, behavioral neuroscientists have been captivated by the prospect of brain “reward circuits.” During the intervening years, a strong case has been made for the nucleus accumbens (NAc) and NAc-projecting dopamine neurons in the ventral tegmental area (VTA) as being critical cogs in brain reward circuitry. Although the precise relationship between dopamine and reward is still under debate (Berridge and Robinson, 1998; Wise, 2004; Salamone, 2007; Redgrave et al., 2008; Beeler et al., 2012), it is clear that NAc and dopamine participate in processing rewarding stimuli and the generation of reward-directed actions (Schultz, 2000; Kelley et al., 2005; Fields et al., 2007; Kenny, 2011). With respect to dopamine neurotransmission, there is robust agreement using a variety of tools, that the majority of dopamine neurons increase their firing rate and dopamine concentration increases in NAc in response to unpredicted primary rewards or cues that reliably predict rewards (Schultz, 1998; Roitman et al., 2004; Matsumoto and Hikosaka, 2009; Cohen et al., 2012; McCutcheon et al., 2012). However, there has been comparatively little attention paid to dopamine responses to aversive stimuli. Although it is increasingly recognized that the NAc and dopamine process aversive stimuli, the manner in which such stimuli are encoded by this system remains unclear.

Our behavior is potently modified by both beneficial and harmful outcomes. Inappropriate affective responses are hallmarks of many psychiatric disorders including depression, bipolar, and other mood disorders. For example, in animal models drug-addicted rats will continue to respond for drug even if they must simultaneously endure a foot shock that would normally be considered aversive (Deroche-Gamonet et al., 2004; Vanderschuren and Everitt, 2004). These findings are proposed to be analogous to the insensitivity of human drug addicts to the costs associated with continued drug seeking and taking. While there has been a traditional focus on dopamine and the NAc in behavior associated with beneficial outcomes, it is imperative to gain a further understanding of the nature of NAc-dopamine signaling in aversion and to determine whether these components play as strong a role in rejection responses and avoidance learning as they do in appetitive responses and approach learning.

## Definition of Aversion

Like reward (Berridge and Robinson, 2003), aversion is a multi-dimensional construct. Most aversive stimuli are intensely disliked and will motivate avoidance. However, it is important to note that dislike and avoidance are not synonymous. As such, dislike is a hedonic evaluation and is common to all aversive stimuli (Kravitz and Kreitzer, 2012). In other words, to be considered aversive, experience of the stimulus should induce a negative hedonic state. However, this definition is problematic as monitoring an animal’s hedonic state is difficult, and in some cases impossible. When using taste stimuli the well-established method of taste reactivity (Grill and Norgren, 1978) has been used to quantify hedonic evaluation in human and non-human subjects alike (Berridge, 2000; Steiner et al., 2001). In contrast, for other sensory modalities such an evaluation is more difficult to quantify. Emission of ultrasonic vocalizations (increase in 22 kHz or decrease in 50 kHz) is thought to be related to hedonia (Knutson et al., 2002) but the utility of this method in assessing hedonic state over a wide range of situations has not been comprehensively validated. Thus, due to the difficulty in assessing hedonic state, in many studies of aversive stimuli, avoidance is used as a proxy for aversion.

When considering the concept of avoidance there are important differences between the production of a behavior that avoids an aversive event (negative reinforcement) and suppression of a behavior that would lead to an aversive event (absence of punishment). Additionally, omission of an expected reward, disappointment, and subsequent extinction of behavior, can also be dissociated from aversion as defined here, and these events may invoke a different set of learning mechanisms (Redish et al., 2007). These distinctions between psychological constructs (hedonic evaluation, reinforcement, punishment, and disappointment) are important as they are likely sub-served by distinct processes at both the systems and cellular/molecular level (Kravitz and Kreitzer, 2012). Indeed, although there is often good overlap between dislike and avoidance there are instances during which these become dissociated. In summary, aversion and avoidance should not be equated and care should be taken when extrapolating the aversive nature of a particular stimulus from its ability to generate or suppress behavior.

Importantly, modulatory factors including motivational state and learning from previous experience can also have powerful effects on the hedonic evaluation of a stimulus. A striking example of motivational state affecting stimulus evaluation is that of salt appetite. Normally, hypertonic sodium chloride solutions are perceived as aversive and unpalatable. In times of need, however, such as following sodium depletion, these solutions become rewarding rather than aversive (Berridge et al., 1984; Tindell et al., 2006); this shift in hedonic valence is accompanied by changes in neuronal activity evoked by hypertonic sodium chloride solutions in NAc (Loriaux et al., 2011) and ventral pallidum (Tindell et al., 2006). Likewise, history with a hedonic stimulus can alter hedonic reactions to it when next encountered. Sweet solutions normally evoke positive hedonic responses. However, if the taste of a sweet solution is paired with malaise, a conditioned taste aversion (CTA) can develop and the same solution is now met with negative hedonic reactions. This shift in hedonic valence is accompanied by changes in neuronal activity evoked by sweet solutions in NAc (Roitman et al., 2010) and basolateral nucleus of the amygdala (Kim et al., 2010). The ability of motivational state and learning to radically alter the nature of a stimulus should make us wary of assuming the hedonic value of a stimulus. This is particularly relevant to studies performed in anesthetized animals. By its very nature, the anesthetic agent is likely to have dampened the negative hedonic state and thus removed the contribution of the neural circuits that may be of most importance for the aversive experience. In this light, the study of “aversive” stimuli under anesthesia may be fundamentally flawed and should be interpreted with caution. This topic will be returned to in the following paragraphs.

Finally, the temporal nature of aversion can vary and states such as stress and fear may consist of negative hedonic states which persist for long periods. For our purposes, we will focus on discrete stimuli that occur on a timescale of seconds. Specifically, with respect to stress, although many of the aversive experiences we will discuss have also been described as acute stressors and when used chronically produce stress-like symptoms (e.g., dysregulation of hypothalamic-pituitary axis and associated behavioral phenotypes), we will not discuss these data. Instead, we refer the interested reader to excellent reviews on stress, dopamine, and NAc (Marinelli et al., 2006; Nestler and Carlezon, 2006; Koob, 2008).

Here, we focus on how aversion may be encoded by mesolimbic dopamine and the implications for NAc processing. A possible confound when studying the encoding of aversion is that there is relief when aversion is terminated – which is likely to be rewarding. In human subjects, offset of a painful stimulus increases blood flow to the NAc, indicating that this region is activated by relief (Baliki et al., 2010). Thus, we will focus on electrophysiological and electrochemical recordings with sufficient time resolution to correlate changes in activity with the onset and duration of aversive events. We review data and present novel findings that unequivocally demonstrate that classical brain reward circuitry is also exquisitely sensitive to aversive stimuli.

## Modulation of Dopamine Cell Firing and Release by Reward

Dopamine neurons within the substantia nigra pars compacta (SNc) and VTA project to dorsal and ventral striatum, respectively. In the vast majority of studies made in primate and rodent subjects, during reward-related stimuli – e.g., presentation of primary reward, reward-predictive cues, and during reward-directed actions (Schultz, 1998; Joshua et al., 2008; Matsumoto and Hikosaka, 2009; Cohen et al., 2012) – these neurons show a fairly homogenous response. That is, the majority of dopamine neurons respond to such stimuli and they do so uniformly by exhibiting brief, high frequency increases in firing rate. This pattern of neural activity is likely to cause transient increases in dopamine concentration within the striatum – which has been empirically demonstrated (Garris et al., 1997; Phillips et al., 2003; Venton et al., 2003; Roitman et al., 2004; Sombers et al., 2009; Owesson-White et al., 2012). Indeed, using the electrochemical technique of fast-scan cyclic voltammetry, which can detect fluctuations in dopamine concentration on a timescale similar to electrophysiological changes in dopamine neural activity, it has been repeatedly demonstrated that primary reward and reward-predictive stimuli evoke brief increases in dopamine concentration (Robinson et al., 2002; Phillips et al., 2003; Roitman et al., 2004; Owesson-White et al., 2008; Stuber et al., 2008; Brown et al., 2011; McCutcheon et al., 2012). Voltammetry has excellent face validity for capturing fluctuations in dopamine concentration that result from transient activations and suppressions of dopamine cell firing (Sombers et al., 2009; Owesson-White et al., 2012). Thus, combining the literature in which either electrophysiological recordings from dopamine neurons or electrochemical recordings of dopamine release were made, the population response of midbrain dopamine neurons to rewarding stimuli appears to be a transient increase in activity.

## Modulation of Dopamine Cell Firing by Primary Aversive Stimuli

Relative to the reward literature, there are far fewer examinations of the dopamine neuron response to aversive events. In the studies that have been conducted with aversive stimuli, outcomes are much less uniform than seen when reward-related stimuli are used. As such, aversive events are commonly shown to have both excitatory and inhibitory effects on the firing of midbrain dopamine neurons. These studies are reviewed in Table 1. A clear conclusion on the encoding of aversive stimuli by the firing rate of dopamine neurons is limited by several factors. First, identification of neurons within VTA and SNc as dopaminergic based on electrophysiological characteristics remains somewhat controversial (Ungless and Grace, 2012). Second, responses to aversive stimuli have been characterized in either anesthetized or awake and behaving subjects. As discussed earlier, anesthesia may suppress components of the circuit that, when awake would contribute to the generation of a very different dopamine response (Koulchitsky et al., 2012). Third, a wide variety of aversive stimuli have been used to compare with reward-responses. Aversive stimuli used to date include, shock, air puff, foot or tail pinch, and aversive taste stimuli. These stimuli are transduced along very different sensory pathways. They also differ in their intensities and have been characterized from mildly aversive to noxious/painful. Finally, many studies use cues that have been associated with the occurrence of an aversive event and the cue itself comes to elicits a behavior that protects the animal against the aversive stimulus, e.g., an eye blink. Thus, the heterogeneity in dopamine responses to aversive stimuli to date may represent real heterogeneity among different pools of dopamine neurons (Brischoux et al., 2009; Matsumoto and Hikosaka, 2009; Lammel et al., 2011) but may also reflect the heterogeneity of investigative approaches.

**Table 1:** **Neuronal responses of midbrain dopamine neurons to aversive stimuli**.

Reference	Species	Awake?	Aversive event	Region	Cell ID	Outcome	Comments
Chiodo et al. (1980)	Rat*	No	Air puff to snout	SNc	EP, Ph	49% Increase, 51% decrease	Inhibited cells have a longer waveform; similar results seen in VTA (unpubl.)
Maeda and Mogenson (1982)	Rat*	No	Foot pinch	SNc, VTA	None	28% Increase, 55% decrease	Divided cells into type I and type II; results shown are combined
Kiyatkin (1988)	Rat	Yes^†^	Tail prick	VTA	EP	67% Increase, 26% decrease	
Schultz and Romo (1987)	Monkey	No	Pinch to face, hand, foot, and tail	SNc	EP, AD, Ph	17% Increase, 51% decrease	
Mantz et al. (1989)	Rat	No	Tail pinch	VTA	EP, AD	0% Increase (MA), 65% increase (MC), 11% decrease(MA), 25% decrease (MC)	Ketamine used for anesthesia
Gao et al. (1990)	Rat*	No	Tail shock	SNc	EP, AD	15% Increase, 78% decrease	
Mirenowicz and Schultz (1996)	Monkey	Yes	Air puff to hand, hypertonic saline, aversive cues	SNc, VTA	EP	14% Increase (US), 3–14% increase (CS), 31% decrease (CS)	Avoidance paradigm – aversive event rarely encountered; potential mediolateral gradient
Guarraci and Kapp (1999)	Rabbit	Yes	Aversive cues (predicting shock to pinna)	VTA	EP	29% Increase, 14% decrease	
Ungless et al. (2004)	Rat	No	Foot pinch	VTA	IHC, EP	0% Increase, 83% decrease	
Coizet et al. (2006)	Rat*	No	Foot pinch and shock	SNc	EP	12% Increase, 72% decrease	
Joshua et al. (2008)	Monkey	Yes	Air puff to eye, aversive cue	SNc	EP, Ph	Increase across population to CS and US	
Brischoux et al. (2009)	Rat	No	Electric shock to paw	VTA	IHC, EP	36% Increase, 36% decrease	Dorsal-ventral segregation of responses
Brown et al. (2009)	Rat	No	Pinch or electric shock to paw	SNc	IHC	5% Increase (pinch), 18% decrease (pinch), 0% increase (shock), 20% decrease (shock)	
Matsumoto and Hikosaka (2009)	Monkey	Yes	Air puff, aversive cue	SNc, VTA	EP	37% Increase (CS), 23% decrease (CS), 11% increase (US), 46% decrease (US)	Dorsolateral–ventromedial segregation of CS and US responses
Mileykovskiy and Morales (2011)	Rat	Yes	Aversive cue (predicting tail shock)	VTA	IHC	20% Increase/decrease, 60% decrease/increase, 20% decrease/decrease	Biphasic responses of cells to onset and offset of CS
Wang and Tsien (2011)	Mouse	Yes	Free fall, shake, aversive cues	VTA	EP	25% Increase (US), 72% decrease (US), 50% increase (CS), 50% decrease (CS)	Many cells show rebound excitation at offset of aversive stimulus
Zweifel et al. (2011)	Mouse	Yes	Foot pinch	VTA	Ph, EP	35% Increase, 35% decrease	Similar results in quinpirole-insensitive neurons
Cohen et al. (2012)	Mouse	Yes	Air puff to face	VTA	Opto	12% Increase, 24% decrease	Excitations in 93% of GABA neurons

Percentages are of total “identified” population. *Female; ^†^head-restrained; SNc, substantia nigra pars compacta; VTA, ventral tegmental area; EP, electrophysiological; Ph, pharmacological; AD, antidromic stimulation; IHC, immunohistochemical; Opto, optogenetic; CS, conditioned stimulus; US, unconditioned stimulus; MA, mesoaccumbal; MC, mesocortical.

## Modulation of Dopamine Release by Aversion

Fluctuations in dopamine concentration in dopamine terminal regions overcome some of the limitations of recording neural activity in the ventral midbrain. There is no controversy surrounding the identity of the compound studied when microdialysis or fast-scan cyclic voltammetry are used. Microdialysis, though, lacks the sampling resolution required to resolve changes in dopamine evoked by discrete aversive stimuli. Only a handful of studies have employed fast-scan cyclic voltammetry to measure fluctuations in dopamine concentration evoked by aversion (Table 2) (Roitman et al., 2008; Anstrom et al., 2009; Wheeler et al., 2011; Budygin et al., 2012) and are subject to the issues identified earlier: specifically, stimuli that are not temporally discrete, stimuli that are transduced along different sensory pathways than rewarding stimuli, and studies that are performed in anesthetized animals. Recently, we and others have measured dopamine fluctuations during intra-oral delivery of rewarding and aversive taste stimuli. Intra-oral delivery, when paired with fast-scan cyclic voltammetry, offers several advantages. First, primary taste stimuli can be selected to evoke reliable and stereotypical appetitive and aversive responses which can be quantified using taste reactivity (Grill and Norgren, 1978; Peciña and Berridge, 2000). Second, rewarding and aversive stimuli are transduced via similar sensory machinery – that is, the taste system. Third, the animal’s exposure to a stimulus can be tightly controlled, which is particularly important when studying stimuli, e.g., a bitter solution, that an animal would actively avoid. Thus, in conjunction with fast-scan cyclic voltammetry, dopamine concentration fluctuations on a timescale commensurate with the subject’s sensory experience can be measured. Using different stimuli, we have shown that an appetitive sucrose solution increases while an aversive quinine solution suppresses phasic dopamine concentration fluctuations in the NAc shell subregion (Roitman et al., 2008). Recordings were made in a region identified as a “hedonic hotspot” (Peciña and Berridge, 2005). These effects were replicated and extended to taste solutions that are used as conditioned stimuli. When one flavored sweet solution predicted the delayed opportunity to self-administer cocaine, it acquired aversive properties (Wheeler et al., 2011). This solution also suppressed phasic fluctuations in NAc shell dopamine concentration whereas a differently flavored sweet solution increased NAc shell dopamine. Thus, rewarding taste stimuli increase and aversive taste stimuli suppress phasic fluctuations in NAc shell dopamine concentration – suggesting that reward and aversion both evoke changes in phasic dopamine signaling but in opposite directions. However, in both studies, different taste solutions were compared. Perhaps the most rigorous test of differential encoding of reward and aversion by phasic dopamine would be to use the same stimulus but in each case to change the animal’s hedonic evaluation of that stimulus. We accomplished this using a CTA paradigm. Here, we measured phasic dopamine signaling in the NAc shell during intra-oral delivery of a sucrose solution. However, for half of the rats (Paired), this sucrose solution had been previously paired with a malaise-inducing injection of lithium chloride in a CTA paradigm. This classical conditioning procedure renders the sucrose solution aversive (Roitman et al., 2010) – which we quantified using taste reactivity. As such, responses to an identical taste stimulus can be compared between rats that have undergone the CTA procedure and those that have not (Unpaired).

**Table 2:** **Phasic dopamine responses to aversive stimuli**.

Reference	Species	Awake?	Aversive event	Region	Outcome	Comments
Kiyatkin (1995)	Rat	Yes	Tail pinch	NAc	Increase	Slow time course, e.g., over minutes
Roitman et al. (2008)	Rat	Yes	Quinine infusion	NAc shell	Decrease to stimulus	
Anstrom et al. (2009)	Rat	Yes	Social defeat	NAc core	Increase in transients	
Wheeler et al. (2011)	Rat	Yes	Infusion of cocaine-paired saccharin solution	NAc shell	Decrease to stimulus	
Budygin et al. (2012)	Rat	No	Tail pinch	NAc core and shell dStri	Increase to stimulus	Greater in NAc than in dStri; slow onset in NAc shell
Park et al. (2012)	Rat	Yes	Quinine	dlBNST	Decrease to stimulus	

*NAc, nucleus accumbens; dStri, dorsal striatum; dlBNST, dorsolateral bed nucleus of stria terminalis*.

## Sucrose Differentially Modulates Phasic Dopamine Concentration Fluctuations Depending on Its Hedonic Value

Male Sprague-Dawley rats (Charles River; *n* = 15) were used. Two cohorts were dedicated to the CTA experiment and were divided into Paired (*n* = 5) vs. Unpaired (*n* = 5) groups. A third group received intra-oral infusions of quinine as a comparison (*n* = 5). All rats were singly housed under standard housing conditions. Food and water were available *ad libitum* throughout the experiment. Surgical procedures were identical to Roitman et al. (2008). Briefly, under ketamine/xylazine anesthesia, rats were surgically implanted with intra-oral catheters, a guide cannula directed at the NAc shell, an Ag/AgCl reference wire in the contralateral cortex, and a bipolar stimulating electrode in the midbrain. After recovery from surgery, Paired and Unpaired rats underwent conditioning. Paired rats received 30 intra-oral sucrose infusions (0.3 M; 200 μL; 4 s; 30–90 s inter infusion interval) on Days 1 and 3 followed immediately by an injection of LiCl (0.15 M; 20 mL/kg; i.p.). On Days 2 and 4, this cohort received saline injections (0.9%; 20 mL/kg; i.p.) in their home cages. For Unpaired rats, the procedure was identical except the injection order was reversed so that intra-oral sucrose infusions were followed by saline injections on Days 1 and 3 and LiCl injections were delivered in home cage on Days 2 and 4. Thus, both groups had the same number of sucrose infusions, LiCl, and saline injections, however, Paired rats had sucrose explicitly paired with LiCl whereas Unpaired rats did not. Quinine rats underwent no conditioning sessions. Next, all rats had a carbon fiber electrode lowered into NAc shell and dopamine release was recorded using fast-scan cyclic voltammetry while rats received sucrose (CTA rats) or quinine infusions, under the same schedule as in training. Dopamine concentration was extracted from current-voltage plots using established methods (Heien et al., 2004; Keithley et al., 2010). For CTA rats, 1–5 days after the recording session, taste reactivity to intra-oral sucrose infusions was video taped and movies were scored for positive (tongue protrusions, lateral tongue protrusions), and negative (gapes, forelimb flails, chin rubs) responses consistent with previous reports (Peciña and Berridge, 2000). At the end of the experiment, in all rats, the recording site was lesioned, rats were transcardially perfused and brains were sectioned for *post hoc* histological confirmation of recording placement.

**Figure 1:**
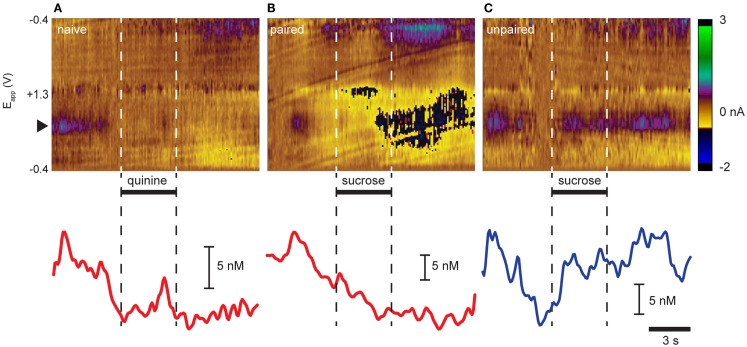
**Opposing effects of aversive and rewarding taste stimuli on dopamine release in NAc shell**. Representative trial examples resulting from intra-oral infusions of **(A)** quinine in naïve rats, **(B)** sucrose in rats that had experienced sucrose explicitly paired with LiCl-induced malaise, and **(C)** sucrose in rats that did not have sucrose paired with malaise. Color plots (top panels) show changes in current (color) at different electrode potentials (*y*-axis) over time (*x*-axis). Dopamine is distinguished by its characteristic oxidation peak (∼0.6 V; black triangle; green/purple feature). Dopamine concentration traces (lower panels) are extracted from above using principal component analysis. Horizontal bars and dashed vertical lines indicate time of infusion.

We (Roitman et al., 2008; Owesson-White et al., 2012) and others (Wightman et al., 2007; Sombers et al., 2009) have reported that phasic dopamine release events occur “spontaneously” without being evoked by any overt stimuli. Here, recordings in the NAc shell captured “spontaneous” dopamine release events (Figures 1A–C). Indeed, as seen in the representative trials in Figure 1, dopamine release events were observed in the seconds prior to intra-oral infusions in examples from all three groups. Intra-oral infusions differentially modulated the frequency with which these events occurred. While quinine delivered to naïve rats (Figure 1A) and sucrose delivered to Paired rats (Figure 1B) suppressed dopamine release events, sucrose delivered to Unpaired rats (Figure 1C) increased their frequency.

**Figure 2:**
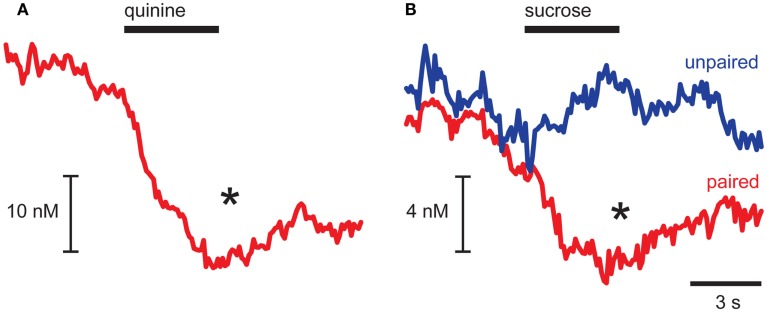
**Aversive stimuli suppress dopamine release in NAc shell**. Averaged dopamine concentration traces showing suppression of dopamine release after quinine **(A)** and sucrose infusions in Paired rats [**(B)**, red trace] and no change in dopamine release in Unpaired rats [**(B)**, blue trace]. *p < 0.05 pre-infusion vs. infusion epoch.

As dopamine release events occurred during the pre-infusion epoch, averaging across trials led to a baseline dopamine concentration from which quinine caused a significant decrease (*p* = 0.032 for pre- vs. infusion epoch; Figure 2A). In CTA rats, sucrose infusions had opposing effects on averaged dopamine concentration relative to the pre-infusion epoch dependent on the conditioning history of the animal (Epoch × CTA interaction, *F*_1,9_ = 7.89, *p* = 0.023; Figure 2B). In Paired rats, which had CTA induced by pairing sucrose with illness, infusions of sucrose caused a significant suppression of dopamine (*post hoc* Tukey’s test, *p* = 0.007; Figure 2B, red trace) similar to what we observed with quinine infusions. In contrast, in Unpaired rats we saw a small increase in average dopamine concentration that was not statistically significant (Figure 2B, blue trace). While in the past we have shown that intra-oral sucrose infusions increase average dopamine concentration in the NAc shell (Roitman et al., 2008), the increase was evoked in naïve rats. Using microdialysis, Di Chiara and colleagues have shown that increases in NAc shell dopamine to novel food reward dissipate with repeated exposure (Bassareo and Di Chiara, 1999). Thus, the weak increase observed in response to sucrose in Unpaired rats may be due to their familiarity with the rewarding sucrose solution.

**Figure 3:**
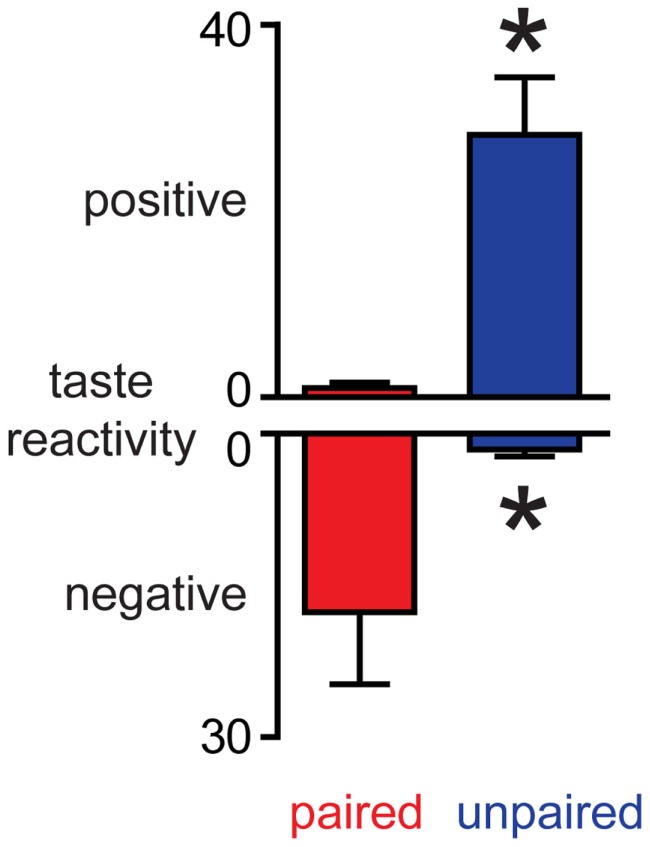
**Induction of conditioned taste aversion leads to a decrease in the palatability of sucrose**. In Paired rats, sucrose infusions evoke more negative and less positive orofacial movements than in Unpaired rats. *p < 0.05 vs. Paired rats.

Conditioned taste aversion rats received a session of sucrose infusions and their orofacial responses were analyzed. In Paired rats, sucrose infusions evoked predominantly negative orofacial movements whilst sucrose evoked predominantly positive orofacial movements in Unpaired rats (Figure 3). These differences were confirmed using Mann–Whitney *U*-tests: paired rats had both higher negative scores and lower positive scores than Unpaired rats (*p*s < 0.05). The data clearly demonstrate that while both groups of rats had equal exposure to sucrose and LiCl, a CTA was established only in Paired rats. Importantly, taken together with dopamine concentration fluctuations, the data establish that in this paradigm, the NAc shell dopamine response matches the hedonic value of the stimulus and, when aversive, the taste stimulus suppresses phasic dopamine signaling.

Electrophysiological recordings from dopamine neurons suggest a heterogeneous response to aversive stimuli – with some studies supporting mostly inhibitory responses (Mirenowicz and Schultz, 1996; Ungless et al., 2004; Cohen et al., 2012) and others supporting the existence of a population of dopamine neurons that are excited by aversive stimuli (Horvitz, 2000; Joshua et al., 2008; Brischoux et al., 2009; Matsumoto and Hikosaka, 2009). Emerging evidence supports anatomical segregation of dopamine neuronal responses (Brischoux et al., 2009; Matsumoto and Hikosaka, 2009; Bromberg-Martin et al., 2010; Lammel et al., 2011) in the midbrain with the conclusion that projection target is a key determinant of each cell’s phenotype and response profile (Lammel et al., 2011). Fast-scan cyclic voltammetry captures fluctuations in dopamine concentration likely caused by phasic changes in electrophysiological activity (e.g., increases and decreases; Garris et al., 1997; Sombers et al., 2009; Owesson-White et al., 2012). As dopamine neurons extensively arborize (Matsuda et al., 2009), cylindrical carbon fiber microelectrodes used for voltammetry likely assay dopamine released from the terminals of different dopamine neurons and thus a net population terminal response. Suppression of phasic dopamine within the NAc shell has now been consistently reported for aversive taste stimuli. This strongly suggests that the population response of NAc shell-projecting dopamine neurons to aversion is that of a decrease in activity.

We have shown here that aversive taste stimuli – those that are innately aversive or acquire aversive properties through conditioning – evoke average decreases in dopamine concentration within the NAc shell subregion. These data replicate (Roitman et al., 2008; Wheeler et al., 2011) and extend previous findings to a CTA paradigm. One difficulty with trying to reconcile studies of reward vs. aversion is that the stimuli used to elicit responses are often qualitatively different and cannot be directly compared. For example, how should an electric shock be treated relative to a sugar pellet? We have circumvented this issue by using taste stimuli, which allow reward and aversion to be studied when stimuli of different hedonic values are conveyed to the central nervous system via the same sensory modality. We deliver solutions directly into the animal’s mouth via intra-oral catheter. Intra-oral delivery gives the experimenter exquisite control over stimulus timing allowing fast neurophysiological or neurochemical events to be correlated with sampling of the stimulus. Furthermore, animals can be exposed to stimuli without requiring a volitional movement thus removing another confound that besets many studies and allowing aversive stimuli that would normally be avoided to be effectively studied.

While we did not assay other striatal dopamine terminal regions, it is possible that responses differ with respect to dopamine terminal locations. Indeed, topographical specificity for responses to reward have been demonstrated (Aragona et al., 2009; Brown et al., 2011; Cacciapaglia et al., 2012). Early studies using microdialysis showed that the dopamine response to foot shock occurs with a greatly different time course in the prefrontal cortex than in the NAc (Abercrombie et al., 1989). Thus, future work will need to consider dopamine terminal sub territories in drawing conclusions about a role for dopamine in both reward and aversion.

## Implications of a Pause in Phasic Dopamine Release in the NAc Shell

Pauses in the electrophysiological activity of dopamine neurons likely underlie the pauses in dopamine release events we observed on single trials and the average decrease, relative to baseline, across trials in which rats experienced aversive taste stimuli. These pauses in dopamine release, in turn, are likely to have their strongest effects on D2 receptor-expressing medium spiny neurons (MSNs). D2 receptors are high affinity (Richfield et al., 1989) and are thought to be mostly occupied even during the asynchronous baseline firing of dopamine neurons that characterizes the absence of salient stimuli (Dreyer et al., 2010). Thus, a pause in dopamine release would lead to a reduction in D2 tone as D2 receptors become transiently uncoupled from dopamine. D2 receptor activation suppresses MSN excitability and the absence of D2 tone causes an increase in excitability (Surmeier et al., 2011). This is particularly interesting because there is strong and growing evidence that NAc neurons, and particularly shell neurons, are excited by aversive stimuli (Carlezon and Thomas, 2009). Tail pinch activates a majority of striatal neurons (Williams and Millar, 1990). Intra-oral infusions of aversive taste stimuli, identical to those used here, evoke primarily increases in the firing rate of NAc neurons (Roitman et al., 2005, 2010), particularly in the shell (Wheeler et al., 2008; Loriaux et al., 2011). In addition, D2 receptor activity has a prominent role in shaping the strength and direction of striatal synaptic plasticity and the absence of D2 receptor tone can shift the balance between long-term depression and long-term potentiation (Calabresi et al., 2007; Surmeier et al., 2011). Thus, pauses in dopamine release coupled with excitatory inputs evoked by aversive stimuli can lead to plasticity in D2 receptor-expressing MSNs and contribute to the learning of appropriate responses to aversive events. The focus on D2 receptor-expressing neurons is especially interesting since their increased activity has recently been shown to be aversive and promotes avoidance learning (Kravitz et al., 2012).

## Potential Mechanisms for Suppressed Phasic Dopamine Release to Aversive Taste Stimuli

Future work must address the mechanisms by which aversive stimuli in general, and taste stimuli specifically, suppress phasic dopamine signaling. Recent publications have focused on this question. Local GABA neurons that suppress the firing rate of VTA dopamine neurons are excited by foot shock in anesthetized rats (Tan et al., 2012) and air puff in awake mice (Cohen et al., 2012). The rostromedial tegmental nucleus (RMTg) is situated just posterior to the VTA, projects to and inhibits dopamine neurons, and is activated by foot shock (Jhou et al., 2009). Neurons within the lateral habenula are activated in response to aversive stimuli, project to the VTA and the RMTg, and contribute to pauses in the firing rate of dopamine neurons (Benabid and Jeaugey, 1989; Matsumoto and Hikosaka, 2007; Stamatakis and Stuber, 2012). It remains unclear, though, how aversive tastes may suppress phasic dopamine release. The parabrachial nucleus, which is the second central relay in gustatory processing, also contains neurons that increase in activity in response to foot shock, project to the VTA, and suppress dopamine neural activity (Coizet et al., 2010). It will be of considerable interest to determine if aversive taste-responsive parabrachial cells project to the VTA and similarly suppress dopamine neural activity. Finally, NAc neurons project, in part, back to the VTA. We have shown that the kappa opioid agonist salvinorin A suppresses phasic dopamine release (Ebner et al., 2010). Since NAc neurons are mostly excited by aversive taste stimuli, dynorphin release leading to kappa receptor activation remains a strong possibility as well.

## Conclusion

Here, we have reviewed literature and presented novel findings detailing the effect of brief aversive stimuli on the neuronal responses of midbrain dopamine neurons and dopamine release in terminal regions. Our data show that in one of these projection sites, NAc shell, the response to aversive stimuli is uniformly a suppression of spontaneous dopamine release. Importantly, the stimuli used were presented in the same modality as rewarding stimuli, which evoke increases in dopamine release. Future work will determine whether these patterns hold true for other projection regions.

## Conflict of Interest Statement

The authors declare that the research was conducted in the absence of any commercial or financial relationships that could be construed as a potential conflict of interest.
